# Reproductive Diseases and Disorders of Female Camels: An Assessment and Pathological and Bacteriological Study in Eastern Ethiopia

**DOI:** 10.1155/2021/6641361

**Published:** 2021-02-13

**Authors:** Dinaol Belina, Amare Eshetu, Sisay Alemu, Bekyad Shasho, Tajudin Mohammed, Ahmedin Mohammed, Bahar Mummed, Dereje Regassa

**Affiliations:** ^1^Haramaya University College of Veterinary Medicine, P.O. Box 138, Dire Dawa, Ethiopia; ^2^Jimma Rare Livestock and Fishery Office, Horro Guduru Wollega, Oromia, Ethiopia; ^3^Wollo University School of Veterinary Medicine, P.O. Box 1145, Dessie, Ethiopia

## Abstract

Camels are the most efficient domesticated animals in arid and semiarid areas of the world. In Ethiopia, they are the main livestock kept to sustain the livelihoods of pastoralists, as camels are used for milk and meat production and also for transportation. However, she-camel reproductive diseases are one of the major constraints for camel-producing communities. A cross-sectional study was conducted from November 2018 to December 2019 to identify and characterize pathological lesions and isolate possible bacteria associated with reproductive diseases and disorders in she-camels slaughtered at Dire Dawa and Babille municipal abattoirs. A total of 155 study animals were examined by recruiting all she-camels slaughtered during every abattoir visit. Overall, 562 reproductive organs, the ovaries, oviducts, uterus, and cervix, were examined through observation, palpation, and incision, and the animal- and organ-level pathological lesion prevalence were found to be 29% and 64.6%, respectively. Degenerative changes, inflammatory lesions (endometritis and salpingitis), growth disturbances (e.g., ovarian hypoplasia), and noninflammatory lesions (e.g., noninflammatory edema) were the identified pathological lesions. Occurrences of pathological changes among reproductive organs had differences where significantly the highest proportion (*p* = 0.00) was observed in the uteri. Of the 119 microbiological samples processed, 77.3% were positive for single or mixed bacterial genera, from which 7 different bacterial isolates and 14 other unidentified Gram-negative bacteria were detected. *E. coli*, *Salmonell*a, and *Staphylococcus* spp. were the most frequently isolated organisms with 28.2%, 26.9%, and 12.8% frequencies, respectively. The result of the questionnaire survey showed 74% of the respondents had culled the she-camel at productive age because of poor reproductive performance associated with refused mating, abortion, and repeat breeding (poor conception). On the other hand, a majority of camel herders had poor to no information and access to modern veterinary services; nevertheless, they had good indigenous knowledge on how to manage reproductive abnormalities. Considering the importance of camels in our study area, further research on camel reproductive diseases and abnormalities with wider sample and epidemiology need to be conducted using molecular and hormonal assay techniques.

## 1. Introduction

Camels are the most efficient domesticated animals in arid and semiarid areas of the world. They play a significant multipurpose role in the dry lands of the world. Pastoralists use camels for transporting grain, water, salt, and other goods, as well as for milk and meat production [1]. Camels also play an important role in offsetting protein deficiencies worldwide for the drastic increase of animal protein demand, particularly red meat protein [2]. A study by Tefera and Gebreah [3] in Eastern Ethiopia indicates camels work on average for 16 hours per day, traveling 60 km. They are very reliable milk producers even during the dry season and drought years when milk from cattle and goat is scarce.

Apart from its long-standing association with desert areas, this animal is endowed with a unique ability to produce and reproduce under extremely harsh desert conditions and to subsist on poor desert vegetation and shortage of water [4]. However, camel products do not make up a significant share of the Ethiopian diet, as Christian religion followers in Ethiopia are forbidden to consume camel milk [5] and camel meat [6]. Major camel-keeping societies in Ethiopia are Afar, Somali, Oromo, Kunama, and Irob pastoralists [7] in the lowland areas. This agroecology of camel production also limits the supply and consumption of camel products. In Ethiopia, a majority of camel producers are also reluctant to sell camel milk due to traditional taboos [5, 6]. Hence, these factors and the dominance of other ruminant species over camels have masked the potential contributions of these animals to the national and household economy. As a result, the camels have been neglected, or at least, their importance is underestimated [8].

The problems of reproduction in camel are not extensively investigated as, for example, in bovine. Available studies, however, indicated that, among other constraints, camel diseases including reproductive diseases are the major problems faced by camel-producing communities throughout the East African countries [9, 10]. The  information collected on these problems is derived mainly from questioning the camel owners, slaughterhouses, and very limited clinical and farm observations [11, 12]. Other scholars reported that repeat breeding, refuse mating, and difficulties in the mating processes are common owner's complaints about she-camel reproduction problems [13]. On the other hand, uterine infections, ovulation failure, early embryonic death, fetal loss, and abortion and management errors are the actual causes of infertility in she-camels though the etiopathogenesis of this syndrome is not well documented [11, 13, 14]. Infection of the genitalia during the peripartum period leads to metritis and endometritis with consequent lowering of reproductive efficiency and repeat breeding [15–17].

Therefore, assessing reproductive performance and isolation of bacteria from the reproductive organs and anatomic pathological investigations are critical for the diagnosis and management of poor reproductive performance in animals including *Camelidae*. Furthermore, bacteriological and anatomic pathological investigations are important to identify the abnormality along with its pathognomonic lesions in specific reproductive organs to intervene the conditions challenging reproductive performance. Hence, the main objectives of the present study were as follows:

- To identify and characterize gross pathological lesions and deformities in reproductive organs of female camels slaughtered at Babille and Dire Dawa municipal abattoirs, eastern Ethiopia
- To isolate and identify aerobic bacteria colonizing the reproductive organs
- To assess camel reproduction constraints and owners' indigenous knowledge in managing reproductive problems

## 2. Materials and Methods

### 2.1. Study Area and Population

The study was conducted from November 2018 to December 2019 in selected districts of eastern Ethiopia, including Babille and Dire Dawa municipal abattoirs. Dire Dawa town is located at a distance of 515 km east of Addis Ababa. It is geographically situated between latitude 9°27′ and 90 49′ north and longitude 41^o^ 38′ and 42^o^ 19′ east. It shares the boundary to the south, south east, and south west with the East Hararghe zone of Oromia region and to the north, east, and west with the Shinille zone of Somali regional state. The main climatic divisions of the area are low land and mid highlands. The average temperature of the area is 25.3c^o^, and the annual rainfall of Dire Dawa varies from 440–760 mm, the rainfall pattern being bimodal. Mixed crop and livestock farming system is the mode of agriculture in the region with camels and shoats as a major livestock which highly contribute for the livelihood of the local community in addition to generating hard currency for the country. The camel population of the area is around 5,070 [18, 19].

Babille woreda is one of the districts of Oromia regional state and located on the main road to Jigjiga at 548.7 km from Addis Ababa to the east of Harar and bordered by the Somali region on the south and east and Fedis and Gursum on the west and north, respectively. The district is located at 700 90′ north latitude and 430 00′ east longitude. The total size of the woreda is about 1,325 km^2^. It is divided into 17 kebeles and 42 subkebeles. The district is mainly situated in the kola climate with shortage of rainfall. Babille is characterized by a semiarid climate with an average temperature of 26.5°C with uneven rainfall distribution. The district has a typical pastoral and agropastoral setting of the country.

Cattle, sheep, goats, and camels are the main animals slaughtered at Dire Dawa and Babille municipal abattoirs where the origin of these animals was mainly from different districts of East Hararghe, Dire Dawa, and Somali regional state. In Dire Dawa municipal abattoir, there are two slaughter premises, the Muslim and Christian slaughter premises, but no such clear premises division in Babille abattoir. Camels are slaughtered in Muslim slaughter premises, and unlike the Christian slaughter premises, the Muslim slaughter premises in the slaughterhouse have no clear division of the slaughtering process into stunning, bleeding, skinning, and evisceration. In both slaughter premises, horizontal bleeding on killing floor but a vertical dressing process on the overhead rail procedure was being conducted.

### 2.2. Study Design and Study Population

An abattoir-based cross-sectional study supported with a questionnaire and or interview was conducted on she-camels slaughtered at Babille and Dire Dawa municipal abattoirs. During the study period, on average, 5–10 camels were slaughtered in the abattoirs, of which 2 to 5 were females. An abattoir visit was conducted twice per week (considering a day on which large number of camels were slaughtered) which depends on local market needs. All female camels slaughtered at the abattoirs during every abattoir visit were recruited in the study. The survey was conducted by distributing a questionnaire and interviewing selected camel herders from Dire Dawa, Babille, and Haramaya who had culled or sold camels during the study. Camel owners were interviewed with pretested questionnaires at the local animal market while selling the camel and also at the household level to measure reproductive performance variables and the reason for culling of the camel. Focal group discussion was made with representative camel owners both at the local animal marketing place and at the peasant association (PA) levels.

### 2.3. Sample Size Determination

Since there were no similar previous studies conducted on she-camels in the study areas, 50% expected prevalence was considered at 95% confidence level and 5% precision for sample size calculation [20].

(1) $$\begin{array}{c} N=\frac{1.96^{2}*\text{Pexp}\left(1-\text{Pexp}\right)}{d^{2}}=384. \end{array}$$

However, due to limited number of she-camels slaughtered during the study period at Babille and Dire Dawa municipal abattoirs, only 155 (58 from Babille and 97 from Dire Dawa) female camels were recruited in the study. From this, a total of 562 reproductive organs, the ovary, oviduct, uterus, and cervix (97 *∗* 4, from Dire Dawa and 58 *∗* 3[cervix excluded] from Babille), were used for the pathological study, and 119 bacteriological samples were collected for aerobic bacterial isolation. Besides, fifty households (camel owners) were interviewed, and focal group discussion was also made with owners on female camel reproductive performance and management practices.

### 2.4. Sample and Data Collection

#### 2.4.1. Questionnaire Survey

Designed questionnaires were supplemented to selected camel owners involved in selling-buying camels during the study period at the animal marketing place and household level and PA sites. Data on history of refused mating, repeat breeding (poor conception), parity number, calving interval, dystocia, early embryonic death/fetal loss, and abortion were collected to estimate the camel's general reproductive efficiency. Owners' indigenous knowledge on she-camel reproductive disease (problems) management practices and level of public awareness (trends to use modern veterinary services) on bringing diseased camels to veterinary clinics and use of common breeding bull were also assessed through pretested and designed questionnaires and/or interviews. In case when delegated individuals are involved in camel selling-buying at the local market, the data collectors directed to the primary owner using orientation from delegates and herdmen were interviewed at the household level. In this, the questionnaire was prepared in English and translated to local languages, “Afaan Oromoo” and, “Amharic,” before the survey. Additionally, focal group discussion was also made with camel owner representatives on female camel reproductive problem and management practices.

In the present study, age of each she-camel was estimated by dental examination on the basis of their dental formulas and tartar deposition on the teeth as previously described [21]. However, to estimate the age at first calving, calving interval and age at culling were calculated based information obtained from owners. The camel herders associate calendar with the number of summer seasons since the occasion such as the number of summers since the first calving and the number of summers between the first and the next calving. Accordingly, one summer means one year.

#### 2.4.2. Sample Collection and Procedures

All female camels brought to the abattoir were appropriately examined for the presence of any abnormal signs during antemortem and postmortem; reproductive organs were removed from the carcass and examined for pathological changes. Microbiological samples were separately collected from any of the reproductive organs observed with lesion(s). Accordingly, swab samples were collected using a cotton swab from the margins and within lesions by opening the lesions with a sterile scalpel blade and putting in a 15 ml test tube containing buffered peptone water (BPW) transport media. Samples were then labeled and, on the same day, transported to the Veterinary Microbiology Laboratory of Haramaya University, using an icebox.

#### 2.4.3. Pathological Identification and Characterization

Following microbiological sample collection, detail postmortem examination was conducted through observation, palpation, and incision of the ovaries, oviducts, uterus, and cervix to characterize pathological changes. Each reproductive organ found positive for lesion was fixed with 10% buffered formalin in a large-mouth glass container, labeled, and transported to the Haramaya University Veterinary Pathology Laboratory for further examination and detail characterization. In cases of larger organs, to avoid transportation difficulty, only the lesion part along with enough normal tissue at the margins was sampled and formalin fixed for transportation. In all processes of tissue fixation, 10% formalin was used in, approximately, a 1 : 10 (v/v) tissue to formalin ratio. Gross lesion examination and characterization were performed according to VMTH [22], in which lesion distribution, contour, consistency, texture, shape, size, and color, as well as the extent and nature of the exudate contained upon incisions, were included and recorded on a format prepared for the purpose.

Lesion severity: Due to the complexity and need for flexibility, it is difficult to get a universal (harmonized) grading system for each involved tissue [23]; thus, few modifications were made in the present study. Mann et al. [23], however, stated the grade of severity assigned to a diagnosis should be chosen to reflect a combination of the extent of the process (how many of its subordinate components are present), the distribution (focal to diffuse), and the actual degree of severity. Hence, severity of gross lesions in our study was conducted based on a semiquantitative procedure adopted in cattle and camel tissue [24–27], modifying it to the context of reproductive organ lesions in she-camels. Lesions on each reproductive organ were scored separately using a 4-point (0–4) grading score, where 0 (normal) = no visible gross lesion; 1 (mild) = no gross lesions on the surface and on palpation but small lesions apparent on incising of the organ or one focal lesion covering <30% of the specific organ size; 2 (moderate) = small gross lesions at more than one focus or lesion covering 30–60% of the specific organ size; 3 (extensive) = gross coalescing, multifocal lesions, or a lesion covering 60–75% of the specific organ size; and 4 (severe) = large or multiple lesions covering >75% of the specific organ size.

The reproductive organ lesion scores were assigned after visually evaluating the organs by four investigators individually and then immediately checked by the group for the final score. The diameter (size) of each lesion (morphological change) was measured using a caliper and ruler to calculate the proportion. Cases such as atrophy (hypoplasia) were compared with the size of the corresponding organ with no visible gross lesions measured from the same age group category of the she-camel slaughtered.

Gross characterization of tumor such as lesion, chronic or acute nature of lesion, and exudate types was supported by cytological techniques. Fine needle aspiration (FNA) and touch imprint slide smears were prepared in duplicates, and one slide was stained with giemsa dye and the other slide was stained with eosin dye. Slides were examined under a microscope, and results were interpreted (lesions characterized) based on the macroscopic and cytological findings by adopting techniques used in a previous study [28]. However, other lesions such as cysts were classified based on their anatomical location and contents.

**Figure 1:**
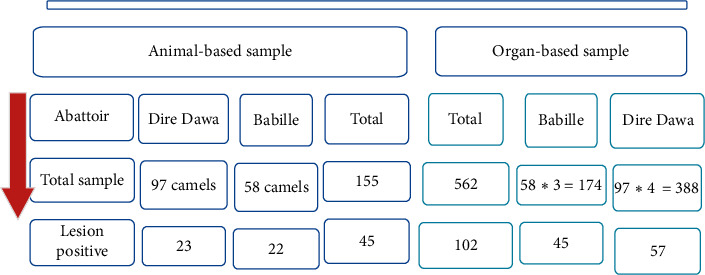
Detail camel- and organ-based pathological sample considered.

The sample size used in the current study considers three targeted assumptions: the abattoir level, animal level, and individual reproductive organ-based categories (Figure 1), and the prevalence results were presented accordingly. In fact, from 45 lesion-positive camels, 23 *∗* 4 plus 22 *∗* 3 = 158, organs were collected; however, at least one organ per animal can be lesion free, and only 102 organs are found lesion positive (Figure 1).

#### 2.4.4. Bacterial Isolation and Identification

All collected swab samples reached the laboratory within six hours from collection time and incubated overnight, and the primary and secondary bacterial isolation and identification were performed according to the work of Quinn et al. [29]. Accordingly, plate cultures were first conducted on general media, blood and nutrient agar, and incubated for 24 hours. Then, based on bacterial growth on culture plate, samples were classified as culture positive (growth observed) and negative (totally no growth). After that, culture-positive samples were examined for bacterial colony morphology, growth patterns, and Gram reaction. Respective colonies were transferred to selective and differential media such as MacConkey agar and selective media such as mannitol salt agar (MSA) and salmonella-shigella agar. In this, Edward's agar medium, Eosin Methylene Blue (EMB), and Xylose-Lysine-Deoxycholate (XLD) were also used for culturing and subculturing of the suspected colonies from the primary test results.

Finally, standard biochemical tests were used for confirmation of bacterial species. Pure colonies were inoculated into Triple Sugar Iron (TSI), Motility Indole Ornithine (MOI), Urease test, and IMViC biochemical tube culture tests as described in the work of Quinn et al. [29]. Specifically, *Enterobacter* and *Klebsella* spp. were differentiated based on their motility test results, whereas the urease test was used to differentiate *Proteus* from *Salmonella* spp. In the present study, bacteria which were culture positive and found Gram negative but which could not grow on some other primary and/or secondary biochemical test cultures we had used were grouped as unidentified Gram-negative bacteria.

#### 2.4.5. Data Management and Analysis

Recorded data were coded and entered into Microsoft Excel spreadsheet and analysed using SPSS version 20.0 software. Descriptive statistics was used to calculate prevalences, and the Pearson chi-square (*χ*^2^) test at a significance level of 5% and 95% CI was considered and observed differences were considered statistically significant at *p* < 0.05. Summary results were presented with tables and graph, whereas lesion characterization was described in pictures and narrative statements. Focal group discussion results were also described in narrative.

## 3. Results and Discussion

### 3.1. Questionnaire Survey

In the current study, to assess female camel reproductive performance, animal dynamic variables such as parity number, age at the first calving, calving interval, age at culling/selling the camel, previous history of reproductive diseases, or problems such as abortion and repeat breeding were considered as factors which might be associated with culling of she-camels and analysed by a questionnaire survey. Under normal conditions, female animals including she-camels are not recommended for slaughter at their productive age, but our study showed that, in every abattoir visit, on average, 5–10 camels were slaughtered, of which 2 to 5 were females. Studies from other parts of Ethiopia also reported female camels kept for production and mature (breeding) females are dominant in the camel herd composition [30, 31].

Majority (70%) of participants in the current study mentioned that age at the first calving in a camel was greater than five years, which is an indication of poor reproductive performance. Comparable findings are also reported in previous studies [7, 30, 31]. The calving interval of camels in our study also varies from 18 to 36 months; however, a majority of the respondents agreed that camels had given birth within 24–30-month intervals. This finding is in partial agreement with reports of previous studies conducted in Ethiopia [21, 31]. In the present study, 10% of our participants mentioned camels had given birth within about 3-year intervals which is contrary to previous reports from Borana [32] and Afar [7, 33], Ethiopia, who reported 12–36- and 31.2-month calving intervals, respectively. Our study result also indicated a significant number (58%) of the respondents used to cull she-camels when the camels were within third to fifth parity number (*p* = 0.02); however, 12% of the herders had culled even before the first calving (Table 1).

The variation in age at the first calving and calving interval of she-camels might be due to differences in the nutritional status of the animal and management practices. For instance, limited availability of veterinary service, high incidence of disease, and poor quality, as well as scarcity of feed and water, affect the age at the first calving. According to Al-Metiery and AI-Hashemy [34] and Ali et al. [35], under improved conditions of nutrition and management, female camels will grow faster and able to reach age at puberty earlier than those kept with scarcity of feed and veterinary services. Use of veterinary services enhances sexual maturity and subsequent productivity by reducing the age at the first calving [30]. The variation in calving intervals might also be due to intentional avoidance of mating, where some camel herders in Ethiopia intentionally avoid mating owing to its negative effect on milk yield, lactation length, and calf survival rate. Inadequate body weight resulting from low plane of nutrition can be a possible cause of delayed age at the first calving in camels [36]. In general, seasonal feed availability, long lactation anoestrus, and infectious diseases are among the factors that contribute to prolonged calving intervals in camels [30, 37].

Attributing to the present study results, a previous study from Jigjiga, Ethiopia, also reported that she-camels are sold (culled) at their productive age [31]. According to Mirkena et al. [7], female camels can remain fertile up to an age of 25 years, during the time which they may produce 8 to 10 calves in a lifetime and, even in average, 11.7 calves are reported from a single she-camel [38]. In general, of the 50 camel owners interviewed, 68% (34/50) had sold she-camels at productive age because of poor reproductive performance. Among the specific reasons mentioned for culling of female camels at reproductive age, refused mating, abortion, and repeat breeding (poor conception) cases accounted 10%, 8%, and 4%, respectively. In contrast to the current study, a majority of herders sell she-camels to obtain income and to buy replacement animals and only 8% of she-camels were culled due to diseases in the Jigjiga zone [31]. Animals with reproductive problems and low milk production are usually culled. To minimize these losses, important disorders of genital organs and their incidence must be defined [39, 40].

Majority (78%) of the camel owners had poor to no information on utilization of artificial insemination (AI) in camels, and only 6 (12%) of the respondents had good information about modern veterinary services and communicated with veterinarians in handling and treating she-camel reproductive diseases or abnormalities. Our study also depicted that 78% of the respondents had used a common breeding bull with their neighbors (natural breeding) and have no knowledge about the direct and indirect impacts of the use of a common breeding bull on she-camel reproductive health or reproductive performance. However, unlike the results of the current study, a previous study report from Somali, Ethiopia, indicated that camel herders had kept 1–3 breeding bulls per camel herd [31]. In contrast to the current study results, use of modern veterinary services significantly enhances milk yield and age at the first calving in camels [30]. Moreover, the results of the questionnaire and interview of the current study indicated that camel owners used to manage animals' reproductive health problems (e.g., dystocia) by raw-egg feeding, oral administration, and genital organ lavaging with leaf preparations (medicinal plants such as *Dodonea angustifolia*) and home feeding perceiving that home feeding prevents the affected she-camel from suspected primary causes and other nearby animals.

Overpacking and exposure to locally available toxic plants were mentioned as predisposing factors for reproductive problems such as abortion or still birth; however, 80% of camel herders had no information about the primary causes or predisposing factors of she-camel reproductive diseases or abnormalities (Table 1). Stress due to overpacking of the pregnant camel and other mismanagement practices might also contribute to fetal loss.

During focal group discussion, camel herders mentioned that they had faced different constraints, and reproductive disorders in she-camels are among the diseases they had been experienced with. The owners also raised that they had poor access of veterinary services. However, they mentioned that they had good knowledge on how to manage reproductive abnormalities including manual dystocia (e.g., abnormal presentation) correction. Keeping sick animal isolated, managing bone breakage including the pelvic bone by massaging, drenching medicinal plants to manage uterine infection, and retaining the fetal membrane are common activities practiced by the participants. The participants also mentioned that they have experiences in washing the she-camel lower genital tract with salt solution when they had identified bad odour and discharges from genitalia after abortion or dystocia cases. In line with our study, Purohit et al. [41] also reported manual dystocia correction in camels is successful, especially when cases are presented within 12 hours. However, the action of nonprofessional individuals can cause other complication such as uterine infection and inflammation due to poor sanitary conditions and physical irritation of the genital organ. Moreover, they had knowledge gap about venereal diseases in case of using common bull for breeding and disease transmission from animals to human through direct contact with infected material in bare hands. Similarly, constraints and reproductive disorders comparable to our study report are previously reported [31, 33].

### 3.2. Postmortem Findings  and Lesion Characterization

**Figure 2:**
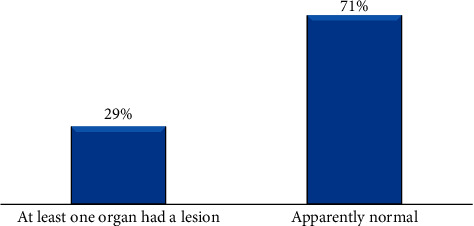
Overall animal-level pathological lesion prevalences in she-camels' reproductive organs

In the current study, for both animal- and reproductive organ-level pathological lesion prevalence determinations, the ovaries, oviducts, uterus, and cervix were separately examined in detail through observation, palpation, and incision. Individual animal was considered positive for pathological lesion when one of the abovementioned reproductive organs were found to have at least a single pathological lesion. Accordingly, animal-level lesion prevalence was 37.9% (22/58) and 23.7% (23/97) at Dire Dawa and Babille municipal abattoirs, respectively. The study also showed that the overall animal-level lesion prevalence was 29% (45/155) (Figure 2).

The animal-level lesion prevalence in our study was higher than the findings of Keskes et al. [42] and Benaissa et al. [43], who, respectively, reported one or more genital abnormalities (pathological lesions) in 19.9% and 28.78% of reproductive organs of female dromedary camels. However, previous reports claimed that camels are resistant to various disease conditions [44]. These variations might arise from differences in the management system and veterinary services, as well as nutrition and physiological differences of the camels at the different study countries [45]. Seasonal variations, age group, breed, status of pregnancy, body condition score, etc. can affect the prevalence of reproductive diseases in female camels. According to Benaissa et al. [43], prevalence of reproductive disorders in she-camels is higher during the winter and summer seasons. The severe hot conditions (which are strongly related to the increase in length of the photoperiod) under which the camel lives directly without any shelter in summer (usually) disturb the physiological functions that affect deleteriously the sexual activity and all the related traits of the camels' polyoestrous nature [46]. In their natural habitats, camels are subjected to severe stress conditions as they are usually raised during the long dry seasons which render them susceptible to many diseases [30, 47]. Beside this, reproductive abnormalities are more common in she-camels less than 10 years of age [43], during which females are at the peak of their reproductive performance with frequent mating, parturitions, postpartum complications, and cervical adhesions. During pregnancy, the uterus is sterile, but after parturition, the uterine lumen is almost always contaminated with a wide range of bacteria. Body condition score (BSC) can also affect diseases with inflammatory processes (metritis, endometritis, cervicitis, and pyometra) [30] though BSC is not considered in the present study.

**Figure 3:**
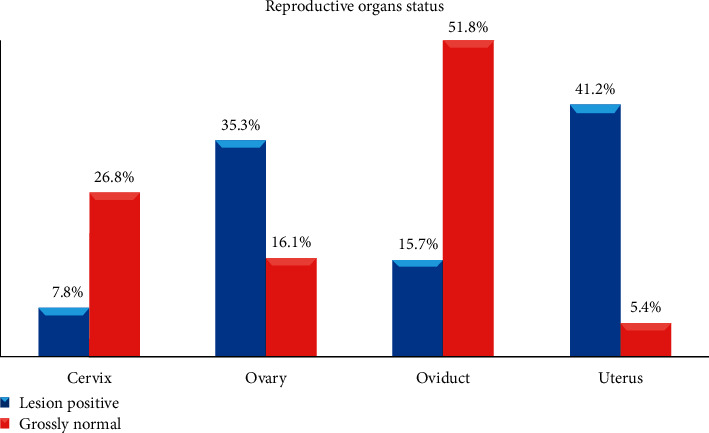
Pathological lesion distribution in reproductive organs of lesion-positive female camels slaughtered at Dire Dawa and Babille municipal abattoirs (*N* = 158 organs).

On the other hand, a total of 158 reproductive organs were collected from the 45 lesion-positive she-camels for detail examination. The result indicated that 64.6% (102/158) of the organs had at least one pathological lesion (Figure 1). The highest prevalence, 41.2% (42/102), was found in the uteri followed by the ovary (35.3%) (Figure 3). This observed organ-level pathological lesion was higher than in previous reports [40] that reported 36.4% pathological lesion.

In the current study, different pathological lesions such as degenerative changes (e.g., deposited materials resulted in bulging of the cervix), inflammatory lesions such as salpingitis, erosive and hyperemic lesions, and growth disturbances (e.g., ovarian hypoplasia), as well as noninflammatory lesions, for instance, tumor such as nodular lesions, and noninflammatory edema and cysts, were examined (Table 2). Occurrences of gross pathological lesions had no differences between female camels slaughtered at Dire Dawa and Babille municipal abattoirs (Table 3); however, variation was found in lesion distribution to the reproductive organs where significantly the highest proportion (*p* = 0.00) was observed in the uterus. Cysts (as active and degenerated form) and calcification were the frequently examined pathological lesions during the study (Table 2).

In agreement with this study, various pathological lesions such as endometritis, pyosalphix, uterine tumor, ovarian hypoplasia, salpingitis, paraovarian cyst, and endometrial congestion with different degrees of severity in reproductive organs of female camels were reported in previous studies [40, 42, 43]. According to the current study, occurrences and distribution of gross pathological lesions varied among reproductive organs of she-camels where significantly the highest proportion (41.2%; *p* = 0.00) was observed in the uterus followed by the ovary (35.3%). The higher incidence of uterine abnormality observed in our study was comparable with the reports of previous studies [11, 40] from Ethiopia and [13] from Saudi Arabia. Uterine lesion including endometritis could be attributed to different factors such as repeated insults of the uterus due to improper mating practices [45], postpartum complications, and unsanitary gynecological manipulations [13, 40].

The present inflammatory lesions in reproductive organs of female camels include endometritis, oophoritis, and salpingitis, in agreement with previous reports [40, 43, 48]. Acute endometritis was macroscopically observed in two uteri as enlarged in size; the mucosa was severely congested (reddish-brown), and in one case, it was severely reddish (supplementary figure 2021-3). In other three she-camels, the lumens of the uteri were thick with blood-tinged exudates. Probably the inflammatory lesions were due to ascending infection and traumatic injury during coitus as herdsmen had practiced manual penis directing into the vagina (intromission) and traditional treatment usually using substances such as dates, black seeds, and salts, which might irritate the mucus membrane leading to inflammation [40]. In agreement to the uterine lesion examined in this study, a higher incidence of the endometritis (45.9%) was reported in Saudi Arabia [13] that uterine infections in camels are mainly associated with acquired reproductive problems and can lead to infertility. Aggressive mating during the “wrong” phase of follicular developmental phase has been reported as a cause of severe uterine inflammation [10, 13, 49]. It is more likely occur after miscarriage or postpartum complications. Various infectious, traumatic, or toxic factors may also results in loss of the ability to resist infection and lead to uterine infections [14]. Management errors such as overuse of males (common bull) can also cause endometritis.

In our study, a tumor such as lesion accounted 11.8% of the total abnormalities examined. In agreement with the present results, tumoral masses are also reported in previous studies [50]. In seven she-camels, the masses were detected as palpable nodules from within (deep) the muscular area of the uterus. Upon incision of the uterine body, small 3 to 5 masses of variable-sized round to oval structures mixed with clotted blood and foci of necrosis with scar like dried were examined. The result of fine-needle aspiration (FNA) and touch imprint slide smear indicated densed cellularity. Variable-sized cells with large nucleus, particularly a heterogeneous population of lymphocytes, were observed, whereas in other three uteri, tumor-like structures were observed as single relatively large palpable masses and firmly attached to the uterine surface. Upon palpation and incision, they were circumscribed, bulging circular nodular masses. Masses of the same gross characteristics with central scars in one of the structures were also observed in 2 ovaries of the studied she-camels. In contrast to this study, ovarian tumors are relatively frequent in animals whereas the tumoral incidence in the oviduct, cervix, and uterine is low [51]. The occurrence and size difference of tumors might suggest hormonal dependencies.

Apart from the small cervix sample considered in this study, various factors such as the structural protective effect of the epithelium and muscle of the cervix can also render it more resistant to infection. Unlike other animals such as cow, the cervix of camels is very short and has four to five distinct rows of annular mucosal folds [48]. The major cervical abnormality encountered was congestion which might be due the problem of vessels draining from the area leading to abnormal accumulation of blood in a part of the congested cervix. In contrast to our finding, closed cervix pyometra is the most prevalent in *Camelidae* and is usually associated with cervical adhesions or prolonged progesterone therapy [52].

Ovarian hypoplasia, different cysts such as ovarian follicular cyst, paraovarian cyst, and oophoritis, are the major pathological changes and disorders recorded in the ovary. Paraovarian cysts are epithelium-lined fluid-filled cysts (Supplementary Figure 2021-1), located near the connective tissue layer attached to the broad ligament between the fallopian tube and the ovary. They were identified in eleven ovaries of she-camels slaughtered. These cysts were observed unilaterally in one side of the ovaries, but in two cases, we examined it from both sides of the ovaries. Follicular cysts were, however, observed in ten ovaries of she-camels (Table 2). These cysts are pale, slightly opaque, and contain clear to straw-colored serous fluid, enclosed in thin layer cells. The walls of ovarian follicular cysts were thin, and cysts were grossly observed as spherical in shape, variable in size, and pale in color. On the other hand, endometrial cysts were identified only in four uteri (Table 2). These cysts are small lump-like structures within the uterus, and they were observed as a fluid-filled structure with yellow to white color.

In the present study, the frequency of paraovarian cysts was higher than that of the ovarian follicular cysts though luteal cysts were not examined at all. Attributing to this, Keskes [11] also reported 3.5% of follicular cysts with no luteal cyst detection. Other studies also reported that occurrence of follicular cysts is higher than that of luteal cysts because luteal cysts originate from luteinization of follicular cysts, which occurred as a result of transformation of the granulosa cells into lutein cells [50]. Moreover, they are often considered to be the later form of ovarian follicular cysts, and therefore, the causes pertaining to follicular cysts can also be considered the original causes of luteal cysts [40, 53]. Though it is difficult to determine the exact cause of ovarian follicular cysts, it can be realized that they develop when one or more follicles fail to ovulate and subsequently fail to regress maintaining growth and steroidogenesis [40, 53, 54]. In partial agreement to our study, in the work of Benaissa et al. [43], ovarian cysts and paraovarian cysts were also reported in higher frequency in she-camels.

The ovarian hypoplastic or atrophic conditions were macroscopically detected as smaller in size and firmer in consistency. They were diagnosed in nine ovaries, as bilateral in three cases and unilateral in the remaining ovaries of the slaughtered camels. In all cases, the hypoplastic ovaries were oval in shape and very small in size, and we could not observe and even palpate the follicles. Such hypoplastic or atrophic ovary conditions were even observed in camels slaughtered during the wet seasons, between April and September. Attributing to the present study, the ovarian hypoplasic conditions in she-camels are also reported in previous studies [40, 42]. However, the size and weight of the ovaries may be affected by the age, size of the animal, and stage of the reproductive cycle. Normally, ovarian size is higher during breeding seasons in young and adult camels [55]. Oophoritis is an inflammatory condition of the ovary. In line with the report of Fathalla et al. [56], oophoritis appreciated with a hyperemic and slightly swollen ovary was found in a very low frequency; however, Mahmoud et al. [57] and Mandefro et al. [40], totally did not report oophoritis from camels.

Erosion and calcification in the oviduct might be associated with upstream extension of a pathogenic organism from the external and then via the uterus, which is also justified by Tibary and Anouassi [14] and Mekibib et al. [58] that untreated uterine infections can lead to irreversible changes in oviducts, thus resulting in sterility due to occlusion. Supporting the report of Rhaman et al. [59], salpingitis (infection and inflammation of the fallopian tubes) and hydrosalpinx (in which the oviduct is filled with an inflammatory fluid) were also observed in the current study (supplementary figure 2021-2). Salpingitis is usually developed from the extension of endometritis or metritis [60].

Examined pathological lesions were characterized for their morphological changes such as distribution, severity, exudate type contained, duration, and size. The study indicated majority of the lesions (48%) were focal whereas 24.5% had soft consistency upon palpation. Different inflammatory conditions, ranging from the self-limited, serous type to the severe granulomatous and fibrinous exudates were mainly investigated in the uterus and oviduct. In our postmortem investigation, 9.8% abnormalities were found either as microlesions or a combination of various pathological changes and considered as “uncharacterized” because we could not characterize them at the gross level. A majority of the lesions observed in the uterus were chronic in nature though acute forms of the lesions were also examined in all reproductive organs: the cervix, ovary, oviduct, and uterus of the female camels (Table 4).

The grade of severity assigned to a diagnosis should be chosen to reflect a combination of the extent of the process (how many of its subordinate components are present), the distribution (focal to diffuse), and the actual degree of severity [23]. In line with this, pathology scoring for the severity of gross lesions was undertaken on tissues, and the result indicated higher frequency of a lesion severity pathology score of 4 was found in the uteri (Table 4). Overall, the severity of reproductive organ lesions showed 48% of the tissue lesions were with moderate pathology (score of 2) and 27.5% of tissues with severe pathology of score 4. This indicates that, in 48% and 27.5% of the examined lesion-positive organs, 30–60% and >75% portions of the organs, respectively, had morphological changes.

In agreement with the report from previous studies [40, 61], suppurative conditions in the present results could be due to bacterial infections and repeated or prolonged exposure to irritant materials. Physical instruments used to aid alternative diagnostic or treatment options can progress lesions into chronic forms such as granulomatous conditions. A granuloma was observed as a focal, compact collection of chronic inflammatory cells, predominantly lymphocytes. It is usually formed as a result of the persistence of a nondegradable product, injurious agent, or chronic irritation of the tissue with attempts of repair. Such lesions were considered as chronic conditions, and they were identified in 51% (52/102) of the lesion-positive organs (Table 4). During the study, for instance, localized granular endometritis (chronic) was observed as multiple pale nodules with white spots of necrosis. The dried necrotic areas of calcification were found hard upon palpation and incision.

The lesions were taken as acute when they are active inflammation with an observation of hyperemic regions. Lesions such as acute fibrinonecrotizing endometritis were identified as the area of coagulative necrosis of the superficial part of the endometrium, while redness (hyperemic) and extensive edema were appreciated below the necrotic layer. Suppurative exudates were grossly observed as pus (creamy yellow) in which the affected tissue is liquefied into a soft viscous mass. A majority of the acute endometritis cases were soft in consistency upon palpation.

### 3.3. Bacterial Species Isolated

In the current study, a total of 119 swab samples were collected from reproductive organs which had gross lesions and processed in the laboratory for aerobic bacterial isolation. Of these samples, 77.3% (92/119) of them were found positive for single and/or mixed aerobic bacterial species. These isolates were categorized in to seven (7) different bacterial species and 14 other Gram-negative bacteria. These Gram-negative organisms did not grow on some other primary and/or secondary biochemical tests we had used; thus, the micro-organisms of such characteristics were considered as *unidentified* Gram-negative bacterial species. Among the 7 identified bacterial species, *E. coli*, *Salmonell*a, and *Staphylococcus* spp. were the most frequently isolated organisms with 23.9%, 22.8%, and 10.9% overall frequencies, respectively. *Klebsella* spp. was found to be the least frequently isolated bacteria (4.3%) from she-camel reproductive organs (Table 5).

On the other hand, of the she-camel reproductive organs sampled, bacterial organisms were most frequently examined from the uterus. The overall frequency of bacterial species in uterine lesions was 66.3% (61/92), of which six isolates were considered as “*unidentified*” Gram-negative organisms. Unlike *E. coli*, *Streptococcus*, and *Proteus* species, the other isolates, *Enterobacter, Klebsella, Salmonella*, and *Staphylococcus* species, were not identified from the cervix (Table 5).

In agreement with the current study, seven bacterial genera were identified from she-camel reproductive organs [42] though salmonella spp. was found to be the second most frequently isolated bacteria in our study. A previous study conducted in Ethiopia also reported *E. coli*, *Staphylococcus*, *Streptococcus*, *Pseudomonas*, *Proteus*, *Salmonella*, and *Klebsiella* species from she-camel reproductive organs [40]. The overall number of identified isolates in the present study was, however, higher than the number of isolates previously reported in she-camel reproductive organs [62]. Such variations might due to a difference in the considered sample size and epidemiological setting of the studies.

Like in other animals, some of the isolated organisms are part of the normal vaginal flora, whereas others are opportunistic and can become pathogenic if the appropriate conditions are present. In our study, the highest proportion of lesion and bacterial isolates were found in the uterus. This could be due to the fact that major bacterial pathogens colonizing the uterine environment contributed to the uterine inflammation and degenerative changes. The number of bacteria colonizing the uterus and the level of uterine immune response are important determinants of uterine infections [63, 64]. When the immune status is lowered, the pathogenic bacteria adhere to the endometrial mucosa, get internalized, and penetrate the epithelium leading to lesion development. Alternatively, the bacteria can also release toxins that cause uterine diseases with different pathological conditions [63, 65]. A previous study also reported uterine inflammatory and degenerative changes are associated with pathogens such as *S*. *aureus* and *E*. *coli* [62].

From an ecological point of view, the ecological niches in the host microflora are not separate environments but are a network of interconnected communities that are continually exchanging [11].Therefore, micro-organisms can enter the reproductive tract from other anatomical sites [66]. The reproductive tract is exposed to trauma and microbial challenges at calving and during the early postpartum period. Most animals will have bacterial contamination of the uterus which is eventually cleared by the immune system in healthy camels.

The findings in the present study have shown that the lesion prevalence in the ovary is higher than the prevalence of bacteria isolated from it. This might be due to the fact that the ovary is less affected by traditional treatment usually using substances such as dates, black seeds, and salts, which might irritate the mucus membrane leading to inflammation. However, Ghoneim et al. [67] explained that bacterial isolates from ovaries may be associated to abrupt manipulation of the ovaries that leads to ascending infections from the uterus. *E. coli*, *Streptococcus* spp., *Pseudomonas,* and *Staphylococcus* spp. are also previously reported from ovaries with inflammatory processes in she-camels [68]. The higher prevalence of ovarian lesion than the bacteria isolated from the ovaries in the current study could also be because of the presence of nonbacterial causes such as cysts accounted for its morphological changes.

In contrast to our study results, Ghoneim et al. [67] reported *S. aureus* and *Klebsiella* spp. as important bacterial isolates from cervical samples. The prevalence of *Proteus* spp. in the current study was, however, higher than that in the report of Ghoneim et al. [67]. Obstetrical manipulation trauma [52] and uterine flushing medium could be a source of bacterial isolates contaminating the cervical region [45]. Generally, our study indicated prevalence of cervical infection is lower than that of the infection of the ovaries, oviduct, or uterus which might be due to good defense action of the cervix mucous-secreting epithelium against bacterial invasion [69].

## 4. Conclusions

Reproductive diseases or disorders directly or indirectly distress animal breeding either by causing infertility or sterility, which leads to heavy economic losses to the livestock owners. In this regard, our study showed that, in every abattoir visit, on average, 2–5 female camels were slaughtered and majority of the camel herders had sold (culled) she-camels at reproductive age because of diseases and poor reproduction. On the other hand, camel owners in our study areas had good indigenous knowledge on how to manage reproductive abnormalities in animals including camels. The detail postmortem examination of the ovaries, oviduct, uterus, and cervix showed 64.6% organ-level pathological lesions with different degrees of severity. The commonly examined reproductive abnormalities include degenerative changes, inflammatory lesions, noninflammatory changes, and other abnormalities such as growth disturbances. A majority of the pathological changes were recorded in the uteri, and they were chronic in nature though acute forms were also examined in all reproductive organs: the cervix, ovary, oviduct, and uterus of the female dromedary camels. On the other hand, seven different aerobic bacteria genera, *E. coli, Enterobacter, Klebsella, Proteus, Salmonella, Staphylococcus*, and *Streptococcus* species, and other unidentified Gram-negative isolates were examined in she-camel reproductive organs.

Therefore, in the abattoir based-study, she-camel reproductive abnormalities need to be regularly investigated using techniques such as ultrasonography at the herd and individual camel level. To explore more about the primary etiology of camel reproductive diseases, further studies that recruit larger sample size in wider epidemiology also need to be conducted employing molecular pathology and microbial sequencing and hormonal assay techniques.

## Figures and Tables

**Table 1:** Owners' knowledge and practices in managing reproductive diseases and reason for culling she-camels at the study area.

Productivity testing criteria		District			Total	(*χ*^2^) *p* value
		Babille	Dire Dawa	Haramaya		
Age at culling	Young	0(0)	6(100)	0(0)	**6(12)**	11(0.02)
	Adult	17(50)	11(32.4)	6(17.6)	**34(68)**	
	Old	2(20)	6(60)	2(20)	**10(20)**	
Age at the first calving	4–5	9(60)	3(20)	3(20)	**15(30)**	6(0.05)
	>5 years	10(28.6)	20(57.1)	5(14.3)	**35(70)**	
	Zero	0(0)	6(100)	0(0)	**6(12)**	
Parity number	1–2	5(50)	3(30)	2(20)	**10(20)**	13(0.04)
	3–5	14(48.3)	11(37.9)	4(13.8)	**29(58)**	
	>5	0(0)	3(60)	2(40)	**5(10)**	
Calving interval	NA	3(33.3)	6(66.7)	0(0)	**9(18)**	8(0.2)
	18–24 months	4(28.6)	8(57.1)	2(14.3)	**14(28)**	
	24–30 months	10(45.5)	6(27.3)	6(27.3)	**22(44)**	
	About 36 months	2(40)	3(60)	0(0)	**5(10)**	
Previous history of RP/D	Abortion	2(50)	0(0)	2(50)	**4(8)**	14(0.03)
	Refuse mating	0(0)	5(100)	0(0)	**5(10)**	
	Repeat breeding	2(100)	0(0)	0(0)	**2(4)**	
	No case	15(38.5)	18(46.2)	6(15.4)	**39(78)**	
Suspected predisposing for RP/D	Malnutrition	0(0)	4(100)	0(0)	**4(8)**	22(0.00)
	Over packing	2(100)	0(0)	0(0)	**2(4)**	
	Premature mating	2(100)	0(0)	0(0)	**2(4)**	
	Toxic plant	0(0)	0(0)	2(100)	**2(4)**	
	Unknown	15(37.5)	19(47.5)	6(15)	**40(80)**	
Use of a common breeding bull	Yes (used)	16(41)	17(43.6)	6(15.4)	**39(78)**	0.7(0.71)
	Not used	3(27.3)	6(54.5)	2(18.2)	**11(22)**	
RP/D management practice	Home feeding	2(15.4)	9(69.2)	2(15.4)	**13(26)**	12(0.05)
	Leaf preparation	0(0)	1(33.3)	2(66.7)	**3(6)**	
	Raw egg feeding	5(50)	3(30)	2(20)	**10(20)**	
	Nothing	12(50)	10(41.7)	2(8.3)	**24(48)**	
Knowledge on artificial insemination (AI)	Know	2(18.2)	9(81.8)	0(0)	**11(22)**	8(0.02)
	No information	17(43.6)	14(35.9)	8(20.5)	**39(78)**	
Contacting vet. for RP management	Yes	3(50)	3(50)	0(0)	**6(12)**	1.2(0.5)
	No	16(36.4)	20(45.5)	8(18.2)	**44(88)**	
Total	**19(38)**	**23(46)**	**8(16)**	**50(100)**		

NA (not applicable) = culled before giving birth or after the 1^st^ parity; RP/D = reproductive problem or disorders.

**Table 2:** Identified pathological lesions and their distribution in reproductive organs of female camels.

Gross Lesion	Reproductive organ (%)				Total
	Cervix	Ovary	Oviduct	Uterus	
Bulged Cervix	2 (100)	NA	NA	NA	2 (2)
Calcification	0	1 (7.1)	4 (28.6)	9 (64.3)	14 (13.7)
Congestion	4 (33.3)	1 (8.3)	1 (8.3)	6 (50)	12 (11.8)
Endometrial cyst	NA	NA	NA	4 (22.2)	4 (3.9)
Edematous	0	0	0	4 (100)	4 (3.9)
Erosive	1 (16.7)	0(0)	3 (50)	2 (33.3)	6 (5.9)
Ovarian follicular cyst	NA	10 (100)	NA	NA	10 (9.8)
Hematoma	0	0	0	1 (100)	1 (1.0)
Hydrosalpinx	NA	NA	1 (100)	NA	1 (1.0)
Hyperemic and or endometritis	0(0)	0(0)	5 (45.5)	6 (54.5)	11 (10.8)
Tumor-like nodule	0	0	2 (16.7)	10 (83.3)	12 (11.8)
Oophoritis	NA	2 (100)	NA	NA	2 (2.0)
Ovarian Hypoplasia	NA	9 (100)	NA	NA	9 (8.8)
Paraovarian cyst	NA	11 (100)	NA	NA	11 (10.8)
Salpingitis	NA	NA	2 (100)	NA	2 (2.0)
Spastic 3rd ring	1 (100)	NA	NA	NA	1 (1.0)
Total	8 (7.8)	36 (35.3)	16 (15.7)	42 (41.2)	102 (100)
(*χ*^2^) *p* value	168 (0.00)				

NA = not applicable.

**Table 3:** Prevalence of pathological lesions in reproductive organs of female camels at Dire Dawa and Babille municipal abattoirs.

Abattoir	Reproductive organ with lesion(%)					(*χ*^2^) *p* value
	Cervix	Ovary	Oviduct	Uterus	Total	
Babille	NA	18(40.0)	7(15.6)	20(44.4)	45(44.1)	7(0.07)
Dire Dawa	8(14.0)	18(31.6)	9(15.8)	22(38.6)	57(55.9)	
Total	8(7.8)	36(35.3)	16(15.7)	42(41.2)	102(100)	

NA = not applicable.

**Table 4:** Grossly characterized pathological lesion in reproductive organs of she-camels.

Lesion characteristics		Organ with lesion				Total
		Cervix	Ovary	Oviduct	Uterus	
Lesion distribution	Diffused	1(4.5)	11(50)	3(913.6)	7(31.8)	22(21.6)
	Focal	6(12.2)	16(32.7)	5(10.2)	22(44.9)	49(48)
	Multifocal	1(3.2)	9(29)	8(25.8)	13(41.9)	31(30.4)

Consistency	Firm	5(11.6)	20(46.5)	6(14)	12(27.9)	43(42.2)
	Hard	2(9.5)	0	4(19)	15(71.4)	21(20.6)
	Normal	1(7.7)	5(38.5)	3(23.1)	4(30.8)	13(12.7)
	Soft	0	11(44)	3(12)	11(44)	25(24.5)

Severity	Extensive	0(0)	0(0)	2(66.7)	1(33.3)	3(2.9)
	Mild	1(4.5)	6(27.3)	5(22.7)	10(45.5)	22(21.6)
	Moderate	4(8.2)	23(46.9)	3(6.1)	19(38.8)	49(48)
	Severe	3(10.7)	7(25)	6(21.4)	12(42.9)	28(27.5)

Exudate type	Fibrinopurulent	0(0)	7(46.7)	4(26.7)	4(26.7)	15(14.7)
	Fibrinous	0(0)	0(0)	1(50)	1(50)	2(2)
	Granulomatous	0(0)	0(0)	4(19)	17(81)	21(20.6)
	Necrotizing	4(36.4)	5(45.5)	1(9.1)	1(9.1)	11(10.8)
	Serofibrinous	1(3.7)	14(51.9)	3(11.1)	9(33.3)	27(26.5)
	Serous	0(0)	1(20)	0(0)	4(80)	5(4.9)
	Suppurative	2(18.2)	0(0)	3(27.3)	6(54.5)	11(10.8)
	Uncharacterized	1(10)	9(90)	0(0)	0(0)	10(9.8)

Size	Atrophied	1(4.8)	8(38.1)	6(28.6)	6(28.6)	21(20.6)
	Enlarged	6(9.7)	24(38.7)	4(6.5)	28(45.2)	62(60.8)
	Mild change	1(5.3)	4(21.1)	6(31.6)	8(42.1)	19(18.6)

Duration	Acute	5(10)	19(38)	9(18)	17(34)	50(49)
	Chronic	3(5.8)	17(32.7)	7(13.5)	25(48.1)	52(51)
	Total	**8(7.8)**	**36(35.3)**	**16(15.7)**	**42(41.2)**	102(100)

**Table 5:** Frequency of bacterial organisms isolated from reproductive organs of she-camels.

Bacterial genera isolated		Frequency of isolates in reproductive organ (%)				
		Cervix	Ovary	Oviduct	Uterus	Total
Bacteria	*E. coli*	1(4.5)	3(13.6)	3(13.6)	15(68.2)	22(23.9)
	*Enterobacter*	0(0)	2(40)	1(20)	2(40)	5(5.4)
	*Klebsella*	0(0)	0(0)	1(25)	3(75)	4(4.3)
	*Proteus*	3(33.3)	1(11.1)	0(0)	5(55.6)	9(9.8)
	*Salmonella*	0(0)	0(0)	1(4.8)	20(95.2)	21(22.8)
	*Staphylococcus*	0(0)	0(0)	3(30)	7(70)	10(10.9)
	*Streptococcus*	1(14.3)	3(42.9)	0(0)	3(42.9)	7(7.6)
	Unidentified	1(7.1)	5(35.7)	2(14.3)	6(42.9)	14(15.2)
	Total	6(6.5)	14(15.2)	11(12)	61(66.3)	92(100)

## Data Availability

Data can be obtained from the corresponding author on request.

## Supplementary Materials

Supplementary Materials Supplementary data (on gross pathological changes, data 2021-1-3) accompany this paper, i.e., available for the reader. Figure 2021-1: ovarian cysts: the paraovarian cysts which are epithelium-lined fluid-filled cysts. Figure 2021-2: hydrosalpinx and salpingitis: in hydrosalpinx cases, the oviduct (fallopian tube) was filled with a watery fluid (edematous), and the inflammatory condition (salpingitis) was appreciated with a hyperemic inflammatory region (reddish). Figure 2021-3: endometritis: inflammatory condition of the inner lining of the uterus which was grossly observed with a hyperemic uterus (reddish in colour) with few whitish spots and enlarged size.
