# Supplementary material for: Reproductive Diseases and Disorders of Female Camels: An Assessment and Pathological and Bacteriological Study in Eastern Ethiopia
Source: Vet Med Int. 2021 Feb 13;2021:6641361. doi: 10.1155/2021/6641361 (PMC8057907; doi:10.1155/2021/6641361)
Supplement: Supplementary Materials — Supplementary data (on gross pathological changes, data 2021-1-3) accompany this paper, i.e., available for the reader. Figure 2021-1: ovarian cysts: the paraovarian cysts which are epithelium-lined fluid-filled cysts. Figure 2021-2: hydrosalpinx and salpingitis: in hydrosalpinx cases, the oviduct (fallopian tube) was filled with a watery fluid (edematous), and the inflammatory condition (salpingitis) was appreciated with a hyperemic inflammatory region (reddish). Figure 2021-3: endometritis: inflammatory condition of the inner lining of the uterus which was grossly observed with a hyperemic uterus (reddish in colour) with few whitish spots and enlarged size. [file 6641361.f1.docx]

Some of gross pathological lesions captured during the study (Supplementary data 2021-1-3)


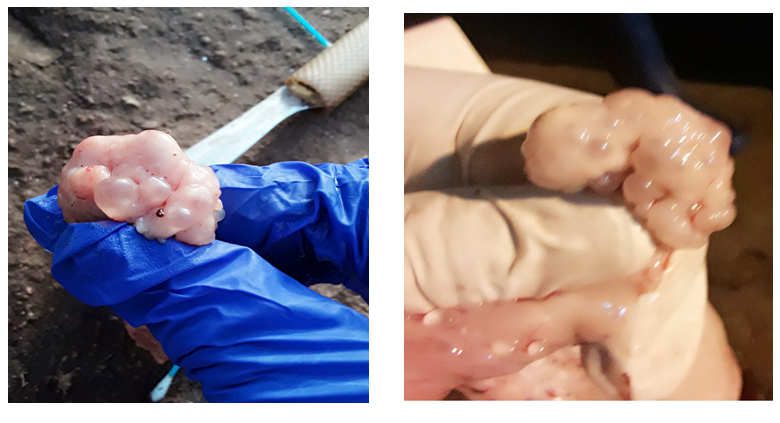


**Figure 2021-1:** Ovarian cysts


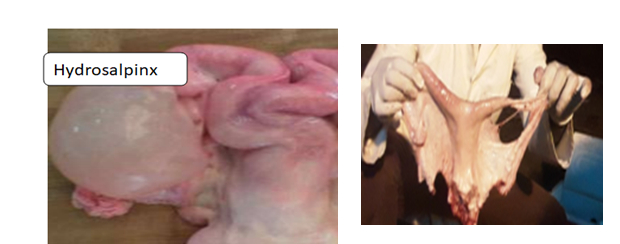


**Figure 2021-2:** Hydrosalpinx and Salpingitis


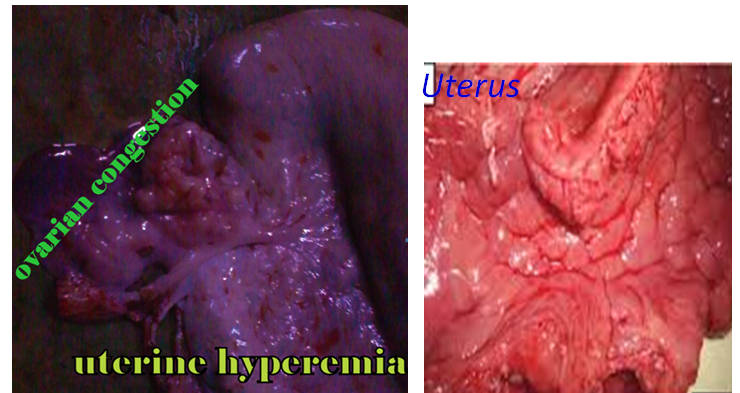


**Figure 2021-3.** Endometritis: Uterine hyperemia and whitish spots

Hydrosalpinx
